# Pyroptosis regulation by *Salmonella* effectors

**DOI:** 10.3389/fimmu.2024.1464858

**Published:** 2024-10-23

**Authors:** Yuan Meng, Qianjin Zhang, Mengen Xu, Ke Ding, Zuhua Yu, Jing Li

**Affiliations:** ^1^ College of Animal Science and Technology/Laboratory of Functional Microbiology and Animal Health, Henan University of Science and Technology, Luoyang, Henan, China; ^2^ The Key Lab of Animal Disease and Public Health/Luoyang Key Laboratory of Live Carrier Biomaterial and Animal Disease Prevention and Control, Henan University of Science and Technology, Luoyang, Henan, China; ^3^ College of Animal Science and Veterinary Medicine, Henan Institute of Science and Technology, Xinxiang, China

**Keywords:** *Salmonella*, pyroptosis, virulence factors, effector protein, inflammasome

## Abstract

The genus *Salmonella* contains the most common foodborne pathogens frequently isolated from food-producing animals and is responsible for zoonotic infections in humans and animals. *Salmonella* infection in humans and animals can cause intestinal damage, resulting in intestinal inflammation and disruption of intestinal homeostasis more severe cases can lead to bacteremia. Pyroptosis, a proinflammatory form of programmed cell death, is involved in many disease processes. Inflammasomes, pyroptosis, along with their respective signaling cascades, are instrumental in the preservation of intestinal homeostasis. In recent years, with the in-depth study of pyroptosis, our comprehension of the virulence factors and effector proteins in *Salmonella* has reached an extensive level, a deficit persists in our knowledge regarding the intrinsic pathogenic mechanisms about pyroptosis, necessitating a continued pursuit of understanding and investigation. In this review, we discuss the occurrence of pyroptosis induced by *Salmonella* effectors to provide new ideas for elucidating the regulatory mechanisms through which *Salmonella* virulence factors and effector proteins trigger pyroptosis could pave the way for novel concepts and strategies in the clinical prevention of *Salmonella* infections and the treatment of associated diseases.

## 1 Introduction

It is well known that when pathogenic microorganisms invade humans and animals, they trigger a host immune response that defends against infection, but host-adapted pathogens employ numerous virulence strategies to overcome these host defense mechanisms. To some extent, the strategies of host-adapted pathogens promote the generation of new host defense mechanisms. At the same time, host defense mechanisms impose evolutionary pressure on the virulence strategies of host-adapted pathogens. Therefore, the interaction between the host and pathogen is a dynamic process that shapes the evolution of the host’s immune response (1). Among the mechanisms that humans and animals utilize to control infections include regulated cell death (RCD) pathways such as pyroptosis, apoptosis, and necroptosis. Over the course of evolution, these pathways have become intricate and complex, coevolving with pathogens that infect hosts. In addition, microbes have evolved strategies to interfere with the pathways of regulated cell death to avoid eradication by the host (2).

*Salmonella* represents a large genus of global public health significance and is the leading cause of foodborne illnesses responsible for thousands of deaths worldwide (3). *Salmonella* has developed multiple strategies to invade and establish a systemic infection in the host (4). For example, these pathogens employ flagella to swim towards the cell and utilize the type III secretion system (T3SS), which allows the translocation of virulence factors or effector proteins into the host for optimal bacterial replication and dissemination (5). Furthermore, the *Salmonella* flagella and T3SS components are recognized by the canonical inflammasome, while lipopolysaccharide (LPS) activates the noncanonical inflammasome formed by caspase-11 in mice (caspase-4 and-5 in humans) (6). Indeed, the inflammatory response is the most prominent outcome of stimulation of innate immune system, which helps to control infection and initiate the development of adaptive immunity (7). As a result, *Salmonella* utilizes a different set of effectors to restrict the inflammatory response to facilitate its survival in the host.

During infection, the death of infected cells and the induction of the immune response are critical for maintaining organismal homeostasis. Programmed cell death (PCD) is a fundamental biological process that plays an essential role in the mammalian host immune defense against invading pathogens (8). Pyroptosis is a novel form of PCD induced by the gasdermin (GSDM) protein family and is accompanied by the secretion of inflammatory cytokines such as IL-1β and IL-18 (9). Pyroptosis can be activated by a variety of extracellular signals, such as extracellular nucleotides, LPS, bacterial DNA and flagellin, as well as intracellular signals, such as oxidative stress, K^+^ efflux, and mitochondrial DNA (10). Subsequently, the GSDM protein family members are triggered to induce pyroptosis by regulating various complex signaling pathways (8, 10) Pyroptosis is specifically induced to eliminate pathogen niches and engage inflammatory responses to potentiate protective host immunity; therefore, it plays an important role in the innate immune response of the host (11). However, growing evidence suggests that bacterial pathogens have evolved to regulate host pyroptosis to evade immune clearance and establish progressive infection.

Recently, based on the interplays between the host and microbes, especially in the case of *Salmonella* infections, increasing evidence has suggested that pyroptosis plays an important role in *Salmonella* infection. This evidence provides increasing insights into the pathogen-driven regulation of pyroptosis and further advances the understanding of the intricate regulatory mechanisms underlying pyroptosis at the host-pathogen interface (8). Here, we describe the latest progress in the study of the mechanisms by which *Salmonella* virulence factors and effector proteins interfere with pyroptosis, which may advance our knowledge of the immune functions and regulatory mechanisms of pyroptosis in the context of bacterial infections. A more in-depth understanding of the regulatory mechanisms underlying pyroptosis-mediated immunity at the host-*Salmonella* interface is helpful for a better understanding of the pathogenic mechanism of *Salmonella* effectors and the complex relationship between *Salmonella* and the host to effectively pave new paths for the rational design of novel vaccines and prevention of *Salmonella* infection in the clinic.

## 2 Pyroptosis

### 2.1 The discovery and definition of pyroptosis

Regulated cell death (RCD), also known as programmed cell death (PCD), is essential for defending against intracellular infections by eliminating the replication niche of pathogens. These pathways include apoptosis, autophagy, necroptosis and pyroptosis, among which pyroptosis is a type of cell death characterized by cell lysis (1). The evolution of human comprehension regarding pyroptosis has been a lengthy journey. Currently, researchers are actively engaged in investigations aimed at acquiring a more comprehensive and profound insight into pyroptosis and the various patterns of cell death.

In 1992, Zychlinsky et al. found that macrophages displayed some of the characteristics of apoptosis such as dependence on caspase when studying *Shigella flexneri-*infected mechanism of action on macrophages (12). Brennan et al. reported that *Salmonella* can induce cell death that is caspase-1-dependent but not accompanied by caspase-3 activation, which is slightly different from the characteristics of apoptosis (13). Subsequently, Boise et al. defined this caspase-1-dependent cell death as “pyroptosis”. “Pyro” means “fire”, indicating that pyroptosis can trigger an inflammatory response, and “ptosis” means “falling”, indicating that pyroptosis is a regulated by cell death (14).

In 2014, Shao, F et al. discovered that LPS can activate the nonclassical pathway of pyroptosis mediated by caspase-4/5/11 in the cytoplasm and further discovered that gasdermin D (GSDMD) of the gasdermin protein family is a key molecule involved in mediating pyroptosis (15, 16). These discoveries have led to a new understanding of pyroptosis. In 2017, researchers discovered that gasdermin E (GSDME) can be specifically cleaved by caspase-3, resulting in oligomerization of the N-terminal fragment of GSDME, thereby inducing pyroptosis (17, 18). Initially, caspase-1 was regarded as a catalyst for cell death. Nevertheless, it has been subsequentially validated that this mechanism is contingent upon the regulation of cell death processes mediated by the GSDM protein family. As research in this domain has advanced, our comprehension of pyroptosis has been significantly enhanced.

### 2.2 Characteristics of pyroptosis

During pyroptosis, the N-terminus of gasdermin family proteins oligomerizes on the cell membrane to form 10-20 nm pores, and then pyroptotic bodies are formed. The cells swell gradually until the plasma membrane ruptures, the cell contents are released, the nucleus condenses, the chromatin DNA breaks, a large number of inflammatory factors are released, and inflammatory reactions occur, ultimately leading to cell death (9).

Regarding the modes of cell death, pyroptosis is usually associated with apoptosis. However, during apoptosis, under the action of caspases, intracellular apoptotic bodies are formed, the nucleus shrinks and becomes fragmented, and DNA is degraded (19). Thus, pyroptosis is different from apoptosis, as the former but not the latter can trigger an inflammatory response.

### 2.3 The activation mechanism of pyroptosis

#### 2.3.1 Inflammasomes: integral components in pyroptosis

In 2002, the Tschopp research team in Switzerland first proposed the concept of the inflammasome (20). The inflammasome is a type of multiprotein complex that is composed mainly of pattern recognition receptors (PRRs), the adaptor protein apoptosis-associated speck-like protein containing a caspase recruitment domain (ASC), and caspases. NLR/ALR family members can contain either a nucleotide-binding domain and leucine-rich-repeat-containing (NLR) protein or an AIM2-like receptor (ALR) protein with an N-terminal PYD. These entities collectively function as PRRs. The PRRs, ASC and caspase-1 form ternary inflammasome complexes through the pyrin domain (PYD) and caspase recruitment domain (CARD) interactions of ASC (21).

Upon activation by pathogen-associated molecular patterns (PAMPs) or damage-associated molecular patterns (DAMPs), both AIM2 and NLRP3 recruit the PYD-containing bipartite adaptor ASC through PYD-PYD interactions. Moreover, ASC can bind to pro-caspase-1 through CARD-CARD interactions, and the three components assemble into complexes to form inflammasomes (22, 23). Currently, the most extensively investigated inflammasomes are NLRP3, NLRC4, and AIM2. As for other inflammasomes, including NLRP1, NLRP6, and so forth, further exploration is required to enhance our comprehension of their functions and mechanisms. Inflammasome assembly can promote the activation of caspase-1, the processing of GSDMD, and the release of the inflammatory cytokines IL-1β and IL-18 precursors, mediating pyroptosis (23). Therefore, the inflammasome plays an important role in pyroptosis and even the innate immune response of the host.

#### 2.3.2 Pyroptosis signaling pathway mediated by the canonical inflammasome

When pathogens infect the host, PRRs located in the cytoplasm are stimulated by danger signaling molecules and recognize PAMPs or DAMPs, including extracellular ATP, pore-forming toxins, RNA viruses, and particulate matter (24). Receptor recognition of pathogens activates PRRs, which bind to ASC. As mentioned previously, the PYD of the adaptor protein ASC connects to upstream PRRs, while the CARD connects to downstream pro-caspase-1 and assembles into inflammasomes such as the NLRP3 inflammasome and the NLRC4 inflammasome (25). The intricate complexes they assemble are capable of activating caspase-1 (26–28). When the inflammasome is successfully assembled, caspase-1 located downstream of the inflammasome is also activated, and GSDMD is cleaved into N-terminal and C-terminal fragments (29, 30). The N-terminal fragment of GSDMD oligomerizes and adheres to the cell membrane, binds to membrane lipids to punch holes in the membrane, and finally leads to membrane rupture and the release of cytoplasmic contents (30). Moreover, caspase-1, which is activated, can promote the maturation and release of IL-1β and IL-18, inducing pyroptosis (26, 30). Therefore, we refer to this pathway, which relies on the activation of caspase-1 by the inflammasome to induce pyroptosis, as the classical pyroptosis pathway (10).

#### 2.3.3 Pyroptosis signaling pathway mediated by the noncanonical inflammasome

Unlike the pathway of caspase-1-dependent pyroptosis, the noncanonical inflammasome pathway of pyroptosis relies on the activation of caspase-4/5/11 (10). The outer membrane vesicles (OMVs) of gram- negative bacteria containing LPS enter host cells through fusion or endocytosis with the cell membrane (31). When host cells are stimulated by LPS, caspase-11 in mice (represented in humans by its direct congeners caspase-4 or caspase-5) can bind directly to LPS (32, 33). The CARD domain of caspase-11 interacts with LPS to form a complex, also known as the noncanonical inflammasome (34, 35). Subsequently, caspase-11 is activated and further cleaves the GSDMD protein. The cell membrane is perforated by oligomerization of the N-terminal fragment of GSDMD. The activation of the noncanonical inflammasome caspase-11 can also induce the proteolytic activation of caspase-1, and activated caspase-1 promotes the maturation and release of the cytokines IL-1β and IL-18, resulting in an inflammatory response and inducing pyroptosis (29, 33, 36).

Following the specific binding of LPS to caspase-11, caspase-11 can break the pannexin-1 channel followed up by ATP release, and then activates the purinergic P2X7 receptor. Cleaving of Pannexin-1 channel and activation of P2X7 can induce the disruption of ion channels in the cell membrane, cytotoxicity and pyroptosis (37). This discovery gives us a new understanding of the signaling events that mediate pyroptosis downstream of caspase-11. The non-classical pathway of pyroptosis characterized by the direct activation of caspase-4/5/11, thereby bypassing the requirement for inflammasome activation.

## 3 A concise overview of *Salmonella*

The *Salmonella* genus represents the most common foodborne pathogens and is a major group of zoonotic foodborne pathogens that cause morbidity, mortality, and disease burden in all regions of the world (38, 39). *Salmonella* is a facultative anaerobic, gram-negative, rod-shaped bacterium belonging to the Enterobacteriaceae family. The *Salmonella* genus is comprised of two species, namely, *Salmonella enterica* and *Salmonella bongori* (40). *Salmonella* bacteria are classified according to their surface structures such as LPS, flagella, and capsule polysaccharides (41). The pathogenesis of *Salmonella* is governed by various pathogenicity islands, secretion systems, virulence genes and effector proteins, which is why these pathogens can cause disease in humans and animals (42). *Salmonella* pathogenicity islands (SPIs) are cluster of genes positioned in a large region of chromosomal DNA that can encode virulence effectors. These SPIs are acquired through horizontal gene transfer (HGT) during bacterial evolution and strongly contribute to the survival, virulence, and dissemination of pathogens.

To date, more than 20 SPIs have been identified, with SPI-1 and SPI-2 having been the subject of extensive research. SPI-1 and SPI-2 are capable of encoding T3SS pertinent to the invasive mechanisms of *Salmonella*, given that these two pathogenicity islands are replete with an array of virulence factors and effector proteins that are integral to bacterial pathogenicity. For instance, genes that encode T3SS translocation proteins, including *sipB*, *sipC*, *sseB*, and *sseC*, genes that encode T3SS effector proteins such as *sopD*, *sopE*, *spiC*, and *sifA*, as well as genes that encode molecular chaperones like *sicP*, *inv*B, *sscB*, and *ssaE*, are all integral to the functionality of the secretion system (42). Pathogenic *Salmonella* employs a sophisticated mechanism involving the T3SS to deliver a suite of specialized effector proteins. These effector proteins coordinate to manipulate numerous signaling pathways within host cells, facilitating the internalization of the pathogen and enabling it to infiltrate non-phagocytic intestinal epithelial cells. This process not only initiates inflammatory responses but also plays a pivotal role in the pathogenesis of *Salmonella* infections.

The remaining SPIs including SPI-3 which is responsible for the uptake of Mg^2+^ and encodes MisL adhesins, SPI-4 and SPI-9 which encode the type I secretion systems (T1SS), as well as SPI-6, SPI-7, and SPI-10 which encode pilin proteins, play a critical role in bacterial invasion, colonization, circumvention of the host immune system’s detection mechanisms, and the persistence within host organisms (43). Currently, an extensive body of literature exists concerning the SPIs and secretion systems of *Salmonella*, and as such, we will refrain from delving into these topics in depth within the context of this discussion.

## 4 Interaction between virulence factors or effector proteins by *Salmonella* and pyroptosis

In this study, we systematically organized and meticulously analyzed the interactions between the virulence factors and effector proteins of *Salmonella*, as well as their implications in pyroptosis (**Table 1**). This will facilitate an in-depth comprehension of the characteristics and pathogenic mechanisms of *Salmonella*, offer fresh perspectives for the identification of new antibacterial targets and vaccine development, thereby laying the groundwork for future therapeutic advancements.

**Table 1:** *Salmonella* virulence effector targeting the host pyroptosis.

Effectors	Localization	Biochemical Activity	Functions	Host Target	Can it cause pyroptosis	Interaction mechanism with pyroptosis	Reference
AvrA	SPI-1	Acetyltransferase	Regulate the inflammatory response, inhibit the activation of caspase-3 and apoptosis	JNK, MAPK,	?		(44–49)
SipB	SPI-1	Translocator	Induce apoptosis by activating caspase-8 and caspase-3	Caspase-4	√	Activate pyroptosis by activating caspase-4 in human intestinal epithelial cells	(50–52)
SopB	SPI-1	Phosphatidylinositol phosphate kinase	Promote the survival of *Salmonella* in macrophages and B cells	Akt-YAP	√	Inhibit the pyroptosis of intestinal epithelial cells	(53–57)
SopE	SPI-1	Guanine nucleotide exchange factor	Mediate changes in the membrane skeleton structure of host cells	Rac-1, Cdc-42	?		(58, 59)
SopF	SPI-1	Target phosphoinositide on host cell membrane	Inhibit the activation of caspase-8 and caspase-3, and promote MLKL phosphorylation	PDK-1, RSK	√	Activate Pyroptosis, Apoptosis, Necroptosis of intestinal epithelial cells	(60, 61)
PrgH	SPI-1		Activate the inflammasome in chickens to regulate the inflammatory response	NLRP3, Caspase-1	?	Unknow if it can activate GSDMD	(62)
HilA	SPI-1	Transcription activator of the OmpR/ToxR family	Upregulation of SPI-1 gene expression promotes macrophage pyroptosis	SPI-1 gene	√	Activate macrophage pyroptosis by promoting of SPI-1 gene expression	(63–65)
QSec	SPI-1	Membrane bound histidine sensor kinase	Regulate the expression of some virulence genes of *Salmonella*	*flhD, flhC, sifA, sopB*	√	Inhibit excessive pyroptosis of macrophages by regulating the expression of some virulence genes	(66)
SseL	SPI-2	Deubiquitinase activity	Suppress NF-κB activation and impairs IκBα ubiquitination and degradation in macrophages	IκBα	?		(67)
SpiC	SPI-2		Participate in the expression of *fliC* Activate MAPK signaling pathway, and SOCS-3 expression Inhibit inflammatory response	MAPK, SOCS-3	?		(68)
SpvB	pSLT plasmid	ADP ribosyl transferase	Inhibit the expression of mtROS and inflammasomes	NLRP3, NLRC4, Caspase-1, GSDMD	√	Induce delayed pyroptosis of macrophages through classical pathways	(69–72)
SpvC	pSLT plasmid	Phosphothreonine lyase	Inhibit macrophage pyroptosis and suppress CXCL-2, CXCL-3, and CXCR-2 released by macrophages	MAPK, NLRP3, Caspase-1, GSDMD	√	Activate the pyroptosis classical pathway through the MAPK signaling pathway	(73–75)
FepE	O-antigen	Encode very long O antigen chain	Encode very long LPS O antigen chain in *Salmonella* Paratyphi A	Caspase-4	√	Inhibit macrophages pyroptosis by suppressing caspase-4	(65, 76)
FliC,FljB	Flagella		Activate NAIP/NLRC4 inflammasome	NAIP/NLRC4	√	Activate macrophages pyroptosis by NAIP/NLRC4 inflammasome	(77–81)
YdiV	Flagella	Negative regulatory factor of FlhD_4_C_2_	Participate in the expression of *fliC*	*fliC*	√	Inhibit macrophage pyroptosis by supressing the expression of *fliC*	(82–85)
SiiD	SPI-4		Inhibit the production of mtROS and the formation of ASC	mtROS	?		(86, 87)
GalE		Encode a UDP glucose-4-epimerase	Inhibit of inflammasome activation in macrophages	NLRP3	?		(88, 89)
DinJ		Encode *Salmonella* Type II Toxin-antitoxin system (TA)	Inhibit of inflammasome activation in macrophages	NLRP3	?		(90, 91)

The symbol “√” indicates that it can cause pyroptosis. The symbol “?” indicates unknown that means we don't know whether it can cause pyroptosis.

### 4.1 *Salmonella* pathogenicity island-related factors and pyroptosis

#### 4.1.1 *Salmonella* pathogenicity island 1-related factors and pyroptosis

*Salmonella* pathogenicity island 1 (SPI-1) is a 40 kb gene cluster that includes 39 genes encoding the T3SS and its molecular chaperones and effector proteins, as well as a number of transcriptional regulators that control virulence gene expression (43). To date, many effector proteins of SPI-1 have been identified in *Salmonella*, and these effector proteins play various roles in *Salmonella* infection, including participating in host cytoskeleton rearrangement, immune cell recruitment, cell metabolism, and the regulation of the host inflammatory response (92). For example, effectors such as InvJ, PrgH, PrgI, and SpaO assemble into the needle-like complex of the T3SS, and SipB, SipC, and SipD transport effectors through this needle-like device, whereas SopB, SopD, and SopE2 induce changes in the actin cytoskeleton, leading to invasion of *Salmonella* (42). Therefore, SPI-1 plays an important role in the interaction between *Salmonella* and host cells (92).

**Figure 1:**
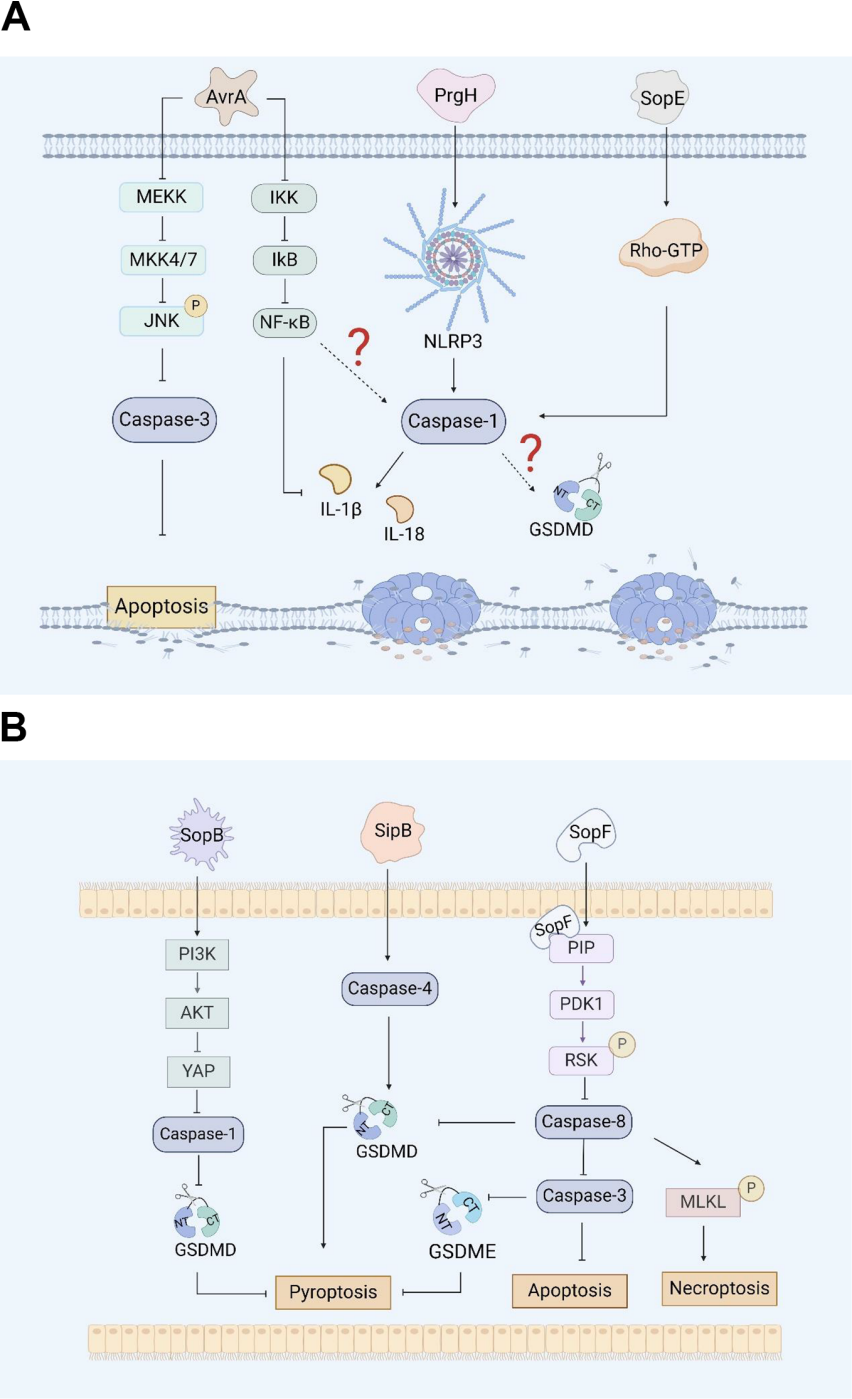
**(A)**. *Salmonella* virulence island 1-related proteins and macrophage pyroptosis. AvrA regulates RAW264.7 macrophage apoptosis through the JNK signaling pathway. PrgH can activates the NLRP3 inflammasome and caspase-1 in chickens. SopE activates caspase-1 in RAW264.7 cells through Rho-GTPase. **(B)**. *Salmonella* virulence island 1 related proteins induce pyroptosis in intestinal epithelial cells (IECs). SopB inhibits Caco-2 cells pyroptosis by inhibiting the AKT signaling pathway. SipB can activate caspase-4 inducing IECs pyroptosis. SopF induces pyroptosis, apoptosis and necroptosis (PANotosis) in Caco-2 cells by inhibiting PDK1-RSK signaling pathway.

AvrA, which is from SPI-1, is a multifunctional protein with acetyltransferase and deubiquitinase activity that inhibits the activation of eukaryotic signaling pathways and host inflammatory responses (44). Research has shown that AvrA effectively blocks the c-Jun N-terminal kinase (JNK) signaling pathway by inhibiting the expression of mitogen-activated protein kinase signaling pathway (MAPK) MKK4/7, thereby inhibiting JNK-mediated apoptosis in chicken and murine models (44, 45). Lin, Z et al. reported that AvrA can inhibit the release of inflammatory factors such as IL-6, IL-18 and IL-1β in mouse intestinal epithelial cells (IECs) through the JNK signaling pathway, thereby inhibiting Caco-2 cell apoptosis (**Figure 1A**) (46). Wang, X et al. reported that the ALK/JNK signaling pathway can activate the NLRP3 inflammasome, inducing pyroptosis during *Streptococcus pneumoniae* infection (47). Zhang, Z et al. reported that GSDME triggered doxorubicin-induced pyroptosis in the caspase-3-dependent manner through the JNK signaling pathway (48). Another study showed that the xanthine oxidase-ROS can activate the MAP3K5/JNK2 substrate licensing complex as a novel regulator of the GSDMD mobilization, which precedes pyroptosis (49). Thus, it appears that AvrA can affect pyroptosis through the JNK signaling pathway. However, this conjecture still requires sufficient evidence to prove.

There are five *Salmonella* invasion protein (sip) genes: *sipE, sipB, sipC, sipD*, and *sipA*. Early studies revealed that the SipB protein of *Salmonella* is similar to the IpaB protein of *Shigella*, which can interact with caspase-1, activate caspase-1, and promote the maturation and release of IL-1β, inducing apoptosis in RAW264.7 macrophages (93). Veronika et al. reported that during the induction of apoptosis by SipB, the activation of caspase-1 during the rapid *Salmonella*-induced apoptosis partially relies on caspase-2 (50). In addition, the caspase-1-independent pathway involves the activation of other caspases and the release of cytochrome from mitochondria, none of which occurs during caspase-1-dependent apoptosis. This means that the protein can activate the apoptosis mechanism by regulating components other than caspase-1 (50). Further research has shown that the SipB protein induces not only caspase-1-dependent cell death but also apoptosis by activating caspase-8 and caspase-3 (50, 51). Other studies have shown that the SipB protein can activate caspase-4, thereby cleaving GSDMD into the N-terminal fragment that oligomerizes, inducing pyroptosis in human intestinal epithelial cells and promoting the maturation and release of IL-18 and IL-1β, which lead to inflammatory reactions (**Figure 1B**) (52). GSDME can be specifically cleaved by caspase-3, which induces pyroptosis, and caspase-1 can interact with caspase-3. However, it is still unclear whether SipB can activate GSDME and cause host pyroptosis through caspase-1 or caspase-3, and this possibility needs to be further explored and verified.

The transcription activator HilA of the OmpR/ToxR family is the central regulator of positive feedback regulation of the SPI-1-encoded T3SS (T3SS-1), which can synergistically regulate SPI-1 genes in response to environmental stress (63). Studies have shown that overexpression of HilA in *Salmonella* Paratyphi A (SPtA) can upregulate the expression of the SPI-1 gene and enhance the invasiveness of *Salmonella* Paratyphi A to host cells, leading to disruption of epithelial cell integrity and promoting the secretion of the cytokine IL-8 (64). It has been shown that overexpression of HilA in *Salmonella* Paratyphi A could promote the invasion of T3SS-1 in THP-1 macrophages and the occurrence of pyroptosis (65).

SopB, also known as SigD, is the main pathogenic protein of *Salmonella* SPI-1 and has inositol phosphatase activity (53). SopB plays an important role in the process of *Salmonella* infection, including the formation of cell membrane folds, the inhibition of fusion of *Salmonella*-containing vesicles (SCVs) with lysosomes, and influencing various cell pathways during the infection process (54). Inflammasome activation plays a crucial role in inducing pyroptosis. SopB promotes the phosphorylation of the Akt-YAP pathway in B cells, inhibits the assembly of inflammasomes, and promotes the survival of *Salmonella* in B cells (55, 56). In addition, SopB-mediated phosphorylation of Akt also inhibits the activation of caspase-1 and GSDMD in Caco-2 cells (**Figure 1B**), thereby inhibiting the pyroptosis, which was conducive to the survival of *Salmonella* in intestinal epithelial cells (57).

The effector protein SopE is a guanine nucleotide exchange factor that can mediate changes in the membrane skeleton structure of host cells, which helps *Salmonella* invade macrophages (54). Early studies suggested that SopE can activate the Rho GTP enzymes Rac-1 and Cdc-42 in host cells, thereby mediating the maturation of caspase-1 and the maturation and release of IL-1β and IL-18 in RAW264.7 cells and preventing the spread of *Salmonella* in the host (**Figure 1A**) (58, 59).

PANoptosis is an inflammatory programmed cell death regulated by the PANoptosome complex and is characterized by essential features such as pyroptosis, apoptosis, and necroptosis. However, PANopotosis cannot be characterized solely by any of the cell death modes of pyroptosis, apoptosis or necroptosis (60). Recent research has shown that the SopF effector of *Salmonella* SPI-1 can trigger Caco-2 cells to undergo PANoptosis. SopF binding to phosphoinositide (PIP) activates the phosphoinositide dependent protein kinase-1 (PDK1) - ribosomal S6 kinase (RSK) signaling pathway to inhibit caspase-8 activation; the activation of caspase-3 and the cleavage of GSDMD and GSDME are downregulated, and the pyroptosis and apoptosis of IECs are inhibited (**Figure 1B**). At the same time, SopF promote the phosphorylation of mixed lineage kinase domain-like protein (MLKL), resulting in programmed necrosis of IECs (61).

The PrgH effector protein encoded by *Salmonella prgH* is an important component of the T3SS-1. Some studies have shown that *prgH* is involved in the activation of the NLRP3 inflammasome, promoting the expression of NLRP3, caspase-1 and IL-1β in chickens, inducing an inflammatory response, and contributing to the invasion and colonization of *Salmonella* Pullorum in the host (62). These findings provide a reference for determining whether *prgH* can induce pyroptosis by promoting the expression of NLRP3, caspase-1 and IL-1β (**Figure 1A**). However, further evidence is needed to fully demonstrate whether PrgH can induce pyroptosis through the canonical inflammasome-mediated pyroptosis signaling pathway.

QSec is a membrane-bound histidine sensor kinase found in gram-negative bacteria that can participate in the regulation of bacterial virulence (66). Studies have shown that QSec blockers can inhibit the expression of QSec-related virulence genes *flhD, flhC, sifA* and *sopB* in *Salmonella* Typhimurium, effectively reduce the virulence of *Salmonella* Typhimurium, and significantly inhibit the excessive pyroptosis of peritoneal macrophages caused by *Salmonella* Typhimurium infection, which is conducive to the clearance of bacteria in macrophages (94).

#### 4.1.2 *Salmonella* pathogenicity island 2-related factors and pyroptosis

*Salmonella* pathogenicity island 2 (SPI-2) is a 39 kb gene cluster that includes the *ssa* operon, which encodes T3SS, the *sse* gene suite, coding for effector proteins, the *ssr* regulatory network, which governs the T3SS expression, and the *ssc* locus, considered to function as a molecular chaperone (95). The T3SS encoded by SPI-2 is also involved in the pathogenesis of *Salmonella*. *Salmonella* transports various effector proteins to the host cell membrane system via the T3SS encoded by SPI-2 and replicates and multiplies within the *Salmonella*-containing vacuole (SCV) (96). *Salmonella* T3SS-2 plays an important role in gastrointestinal diseases and systemic infections, and is also necessary for *Salmonella* survival in different host cells (97).

**Figure 2:**
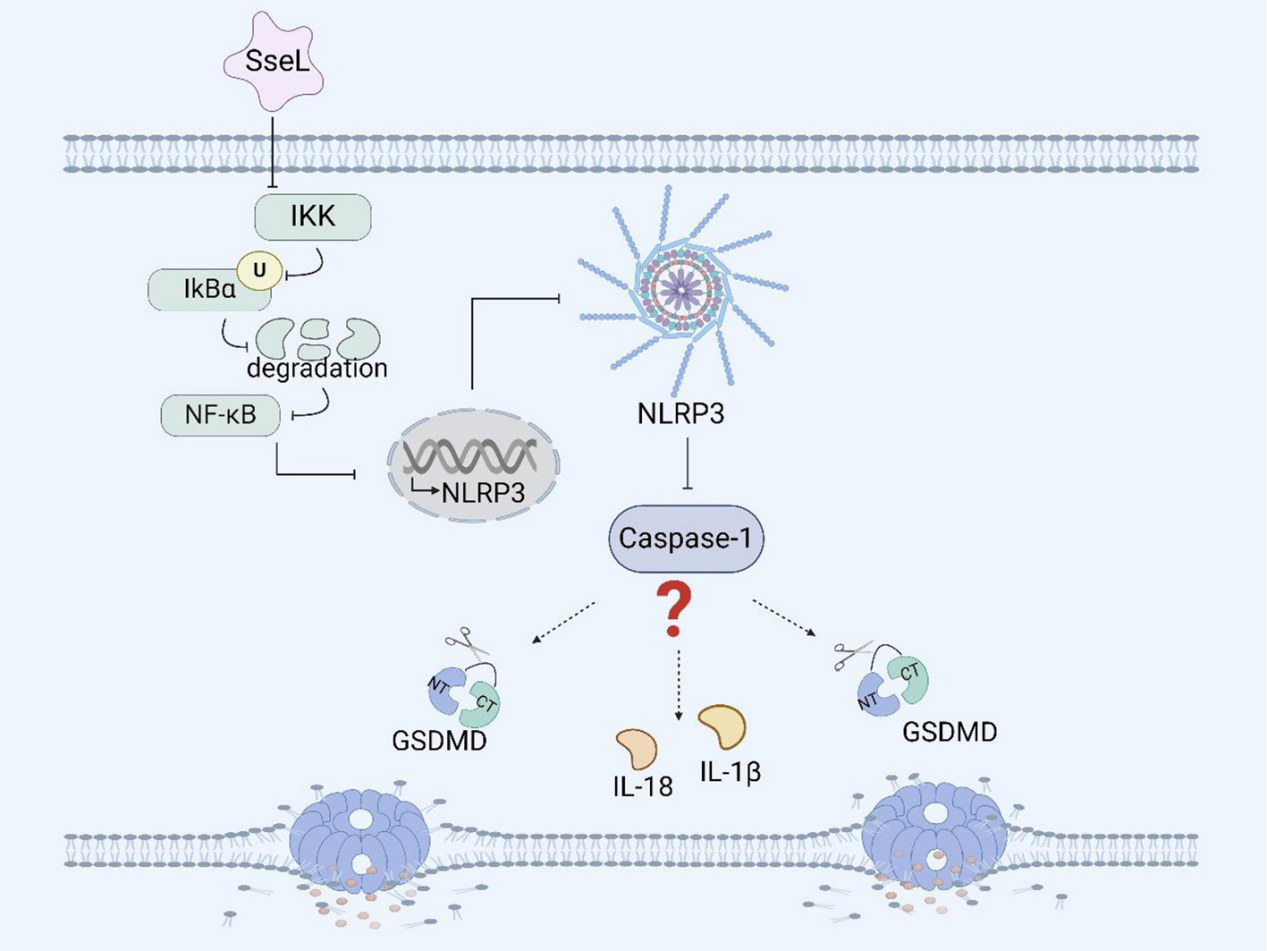
*Salmonella* virulence island 2-related facrors and pyroptosis. SseL inhibits the expression of the NLRP3 inflammasome and caspase-1 in RAW 264.7 cells and BMDMs by inhibiting the NF- κB signaling pathway.

Among many effectors, *Salmonella* secreted factor L (SseL) is a specific protein secreted by the SPI-2-encoded T3SS that can translocate *Salmonella* and is also a putative virulence factor possessing deubiquitinase activity. The expression of SseL in *Salmonella* Typhimurium suppresses NF-κB activation downstream of IκBα kinases and impairs IκBα ubiquitination and degradation in RAW 264.7 cells and bone marrow-derived macrophages (BMDMs), but not IκBα phosphorylation (**Figure 2**) (67). Although the NF-κB transcription factor can promote inflammasome assembly and activation, whether SseL can induce pyroptosis by influencing inflammasome activation through the NF-κB signaling pathway remains to be further investigated (98, 99).

SpiC is a virulence factor encoded within SPI-2. Research has shown that SpiC in *Salmonella* Typhimurium can activate the mitogen-activated protein kinase (MAPK) signaling pathway, restraining the expression of suppressor of cytokine signaling (SOCS-3) in macrophages. The effector SpiC promotes the expression of *fliC* at the transcriptional level (68). FliC is an effector of *Salmonella* flagellar that can activate the NAIP/NLRC4 inflammasome and promote pyroptosis in human and murine macrophages. SpiC can participate in the expression of *fliC*, but whether SpiC affects the occurrence of pyroptosis by affecting the expression of *fliC* is currently unclear and needs to be verified.

In addition, SifA, SpvB, SseF, SseJ, and SteA are all T3SS effector proteins of SPI-2. Studies have shown that these five effectors can synergistically induce cytotoxicity and T3SS-1-independent inflammatory response, but the specific mechanism underlying these effects still needs further research (100).

### 4.2 *Salmonella* plasmid virulence factor and pyroptosis

The *Salmonella* plasmid virulence (spv) genes, including *spvA*, *spvB*, *spvC*, *spvD*, and *spvR*, are ubiquitously distributed across a diverse array of *Salmonella* serotypes. These *spv* genes are integral to the organism’s adhesive properties, invasive capabilities, and its proficiency in surviving within host (101, 102). Studies have shown that SpvA can coordinate the production of virulent proteins in a timely manner and negatively regulate the expression of *spvA, spvB, spvC*, and *spvD* without affecting the expression of *spvR* (103). SpvB can disrupt the integrity of the intestinal epithelial barrier and promote the spread of *Salmonella* throughout the host (104). At present, the interactions between *Salmonella* plasmid virulence genes and pyroptosis that have been studied most frequently involve *spvB* and *spvC.*

**Figure 3:**
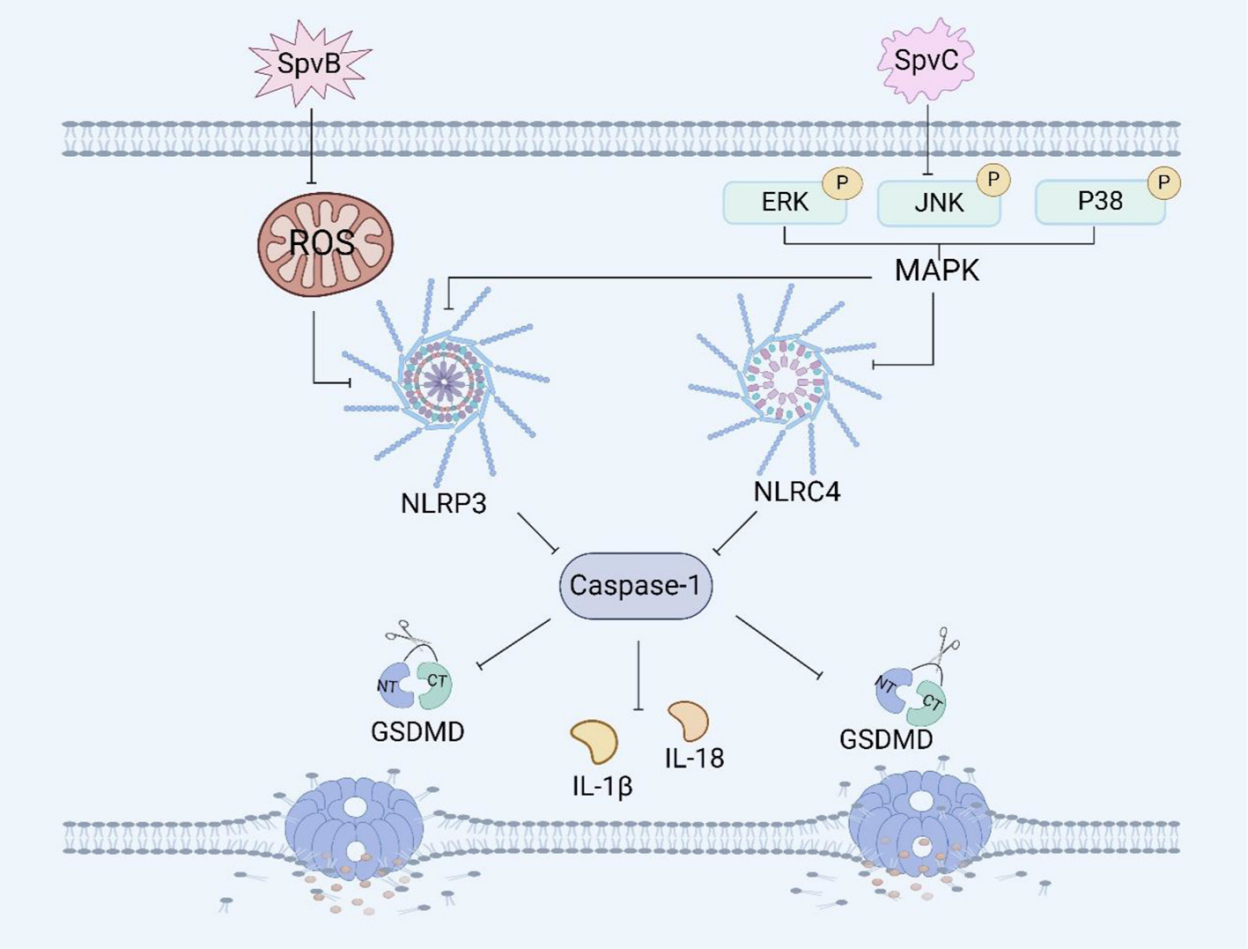
*Salmonella* plasmid virulence factors and pyroptosis. SpvB induces pyroptosis in RAW264.7 macrophages by ROS and the NLRP3 inflammasome. SpvC induces pyroptosis in RAW264.7 macrophages by regulating the MAPK signaling pathway.

The effector protein SpvB has ADP-ribosyltransferase activity, which can reduce the repair ability of DNA and the content of reactive oxygen species (ROS), and ROS can affect inflammasome assembly (69). It has been reported that SpvB can induce necroptosis of IECs by destroying the integrity of the intestinal epithelial cell barrier (70, 104). Recently, We have shown that SpvB can delay pyroptosis in RAW264.7 macrophages (**Figure 3**). The delayed pyroptosis of macrophages induced by the *Salmonella* plasmid virulence gene *spvB* was associated with the influence of the NLRP3 and NLRC4 inflammasomes, and *spvB* inhibited the production of ROS and the activation of NLRP3 at the early stage of infection, and subsequently inhibited the activation of NLRC4 (71, 72).

SpvC has phosphothreonine lyase activity, which can inhibit the intestinal inflammatory response and promote systemic transmission of *Salmonella* through the MAPK signaling pathway (73). It has been shown that the plasmid virulence *spvC* gene of *Salmonella* can inhibit the expression of the macrophage inflammasome components NLRP3 and NLRC4 and the occurrence of pyroptosis in RAW264.7 macrophages through the MAPK signaling pathway (**Figure 3**). Moreover, *spvC* can decrease the levels of neutrophil C-X-C motif chemokine ligand 2 (CXCL2) and C-X-C motif chemokine ligand 3 (CXCL3) released by macrophages, as well as the level of neutrophil C-X-C motif chemokine receptor 2 (CXCR2), and inhibit the release of IL-1β by neutrophils, thereby inhibiting the recruitment of neutrophils and their synergistic antibacterial effect with macrophages (74). In addition, it has also been shown that *spvC* participates in a new mechanism of *Salmonella* pathogenesis and host inflammatory response by inhibiting autophagy and the NLRP3 and NLRC4 inflammasomes (75). SpvC can regulate both autophagy and pyroptosis through the activity of its phosphothreonine lyase, however whether autophagy and pyroptosis can be regulated at the same time and whether the mechanism of their interaction is through autophagy to induce pyroptosis or through pyroptosis to induce autophagy needs further investigation.

### 4.3 Structural virulence factors in *Salmonella* and pyroptosis

*Salmonella* has a complex antigen structure, and serotyping is performed based on the different antigen components on the surface of the bacteria. The antigen components of *Salmonella* include four types: bacterial antigen (O antigen), flagella antigen (H antigen), surface envelope antigen (Vi antigen), and pili antigen (105). Among them, the O antigen and H antigen are the most important antigens and are closely related to the movement, invasion, colonization, biofilm formation and immune escape of *Salmonella* in the host (106).

**Figure 4:**
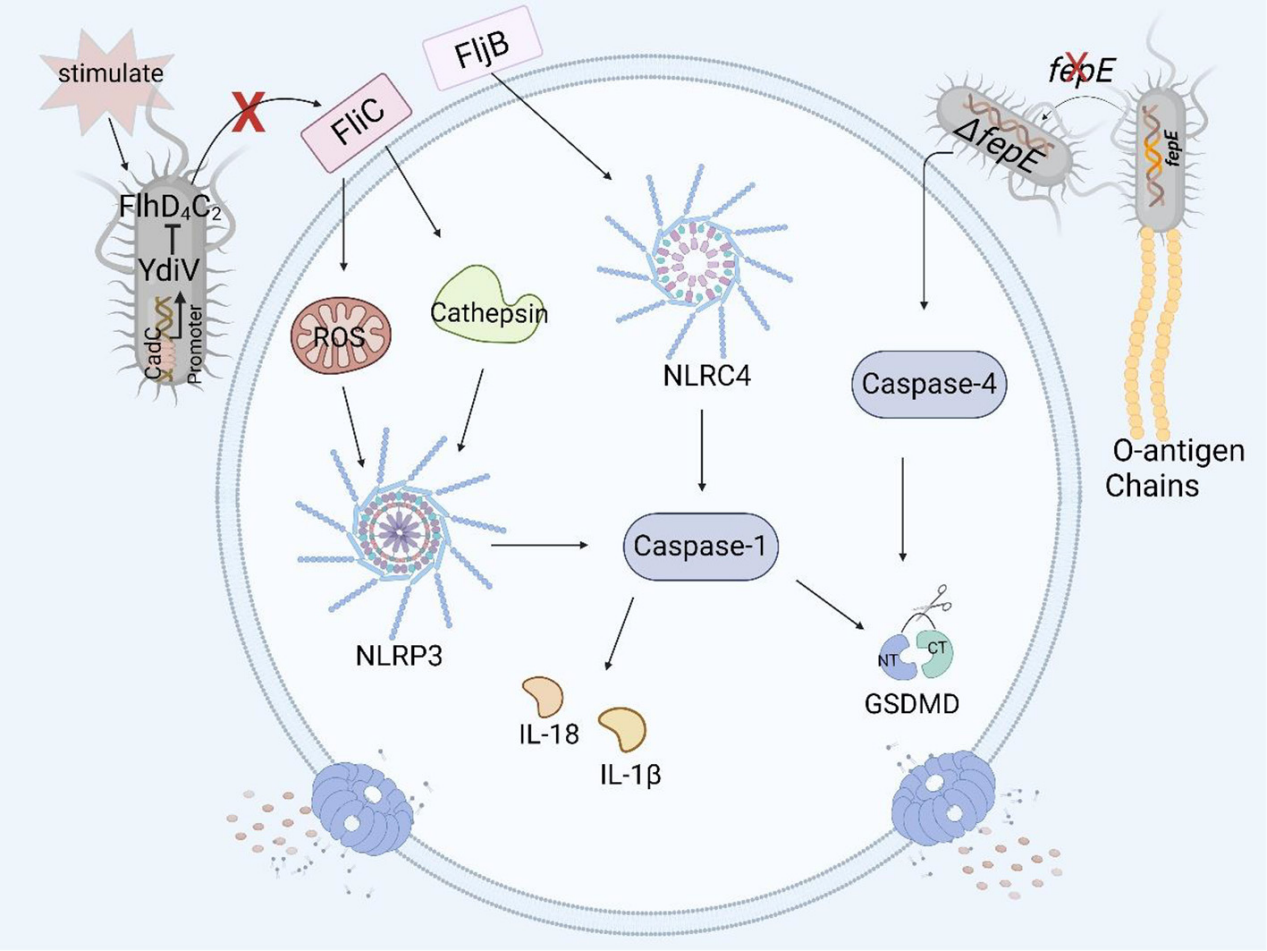
Structural virulence factors in *Salmonella* and pyroptosis. *Salmonella* flagellar protein FliC induces pyroptosis in human and murine macrophages by the NLRP3 inflammasome, but YdiV inhibits the expression of FliC preventing pyroptosis through the FlhD4C2 transposon. FljB induces pyroptosis in human and murine macrophages by activating the NLRC4 inflammasome. The deficiency of *fepE* in *Salmonella* Paratyphi A can activate caspase-4 and induce pyroptosis in THP- 1 macrophages.

*fepE* is a pseudogene in *Salmonella* Typhi, but it can be highly expressed and encode the very long O antigen chain of LPS in *Salmonella* Paratyphi A (76). Researchers have shown that the very long O antigen chain encoded by *fepE* can interfere with inflammasome and caspase-4 activation, inhibit pyroptosis in THP-1 cells (**Figure 4**), and promote immune escape and systemic spread of *Salmonella* Paratyphi A in the host (65).

The flagellin of *Salmonella* is encoded mainly by *fliC* and *fljB* (77). The Nod-like receptor ICE protease-activating factor (IPAF) in macrophages recognizes the flagellin of *Salmonella enterica serovar* Typhimurium and causes the activation of caspase-1 and the secretion of IL-1β (78). In a study of the relationship between flagellin and pyroptosis, it was shown that *fliC* and *fljB* were recognized and assembled by NAIP5 and NAIP6 to form the NAIP/NLRC4 inflammasome. As a result, caspase-1 was activated, and the amino-terminal cleavage fragment of GSDMD induced pyroptosis in mouse macrophages such as RAW264.7 cells and in BMDMs (25, 79). At the same time, the authors also found that a mutation in the *flaA* gene, which encodes a flagellar protein, can lead to the activation of caspase-1 and the occurrence of pyroptosis in macrophages (80). Other studies have shown that the flagellar proteins FliC and FljB activate the NLRP3 inflammasome in THP-1 cells by affecting the production of ROS and the release of cathepsin, thereby activating the downstream caspase family and even inducing pyroptosis (**Figure 4**) (81).

YdiV, a negative regulator of the flagellar main transcriptional activator complex FlhD_4_C_2_, downregulates the expression of the flagellar gene *fliC* through the CadC-YdiV-FlhDC pathway (82, 83). *fliC* can induce pyroptosis in human and murine macrophages, and YdiV inhibits the expression of *fliC*, preventing macrophage pyroptosis and releasing inflammatory factors (**Figure 4**), thereby facilitating the colonization and immune escape of *Salmonella* in host cells (84, 85).

### 4.4 Other virulence factors of *Salmonella* and pyroptosis

**Figure 5:**
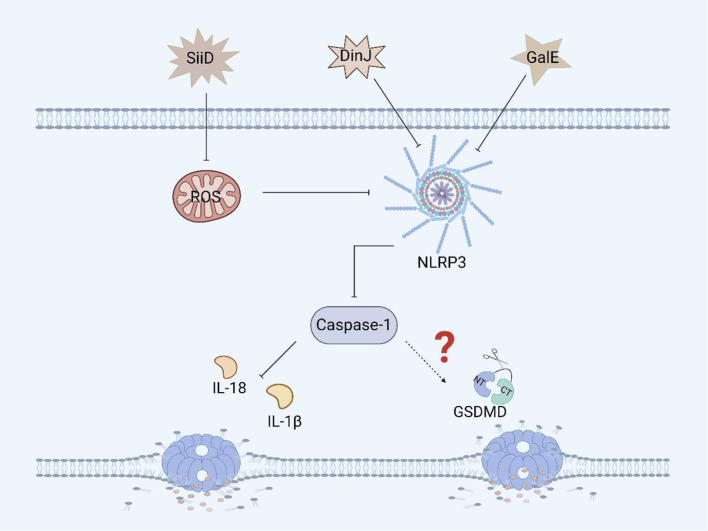
Other virulence factors of *Salmonella* and pyroptosis. SiiD inhibits the expression of caspase-1 in BMDMs by inhibiting the expression of ROS, thereby suppressing the inflammatory response, but it hasn’t yet been proven to inhibit the occurrence of pyroptosis. DinJ can inhibit the expression of the NLRP3 and caspase-1 in BMDMs and J774A.1 macrophages, but further inhibition of pyroptosis remains to be confirmed. GalE can inhibit the expression of the NLRP3 inflammasome and caspase-1 in BMDMs, and whether it can further inhibit pyroptosis remains to be verified.

SiiD, the T1SS effector protein encoded by *Salmonella* SPI-4, further blocks the formation of ASC by specifically inhibiting the production of mitochondrial ROS in BMDMs, thereby inhibiting the activation of the NLRP3 inflammasome, which evades the bacterial clearance mechanism mediated by NLRP3/caspase-1 and promotes *Salmonella* replication and survival, resulting in persistent infection (**Figure 5**) (86, 87). Although we know that SiiD can affect the survival of *Salmonella* in BMDMs via NLRP3/caspase-1, we are not sure whether SiiD induces pyroptosis in BMDMs through NLRP3/caspase-1, and thus, this hypothesis needs to be verified.

GalE encodes UDP-galactose-4-epimerase, an enzyme involved in galactose metabolism and glycosylation (88). GalE can affect the assembly and activation of the inflammasome. A study revealed that *Salmonella* Enteritidis-deficient GalE can activate the NLRP3 inflammasome in mice, promoting the activation of caspase-1 and secretion of IL-1β. These results suggest that the protein can inhibit the expression of NLRP3, caspase-1 and IL-1β in BMDMs and promote *Salmonella* infection (**Figure 5**). Therefore, GalE plays an important role in the regulatory network of *Salmonella* evasion of inflammasome activation. Moreover, the mechanism by which GalE regulates pyroptosis remains to be explored (89).

The DinJ protein encoded by *dinJ* is an antitoxin of the YafQ-DinJ family of the *Salmonella* type II toxin-antitoxin (TA) system (90). The DinJ protein is not only a bacterial antitoxin but also an effector protein that can inhibit the activation of the host inflammasome to evade host defense and promote *Salmonella* infection. A study revealed that DinJ deficiency in *Salmonella* Enteritidis can activate caspase-1 and the secretion of IL-1β and IL-18 in J774A.1 macrophages and BMDMs. Further studies have shown that the DinJ protein can specifically inhibit the activation of the NLRP3 inflammasome, thereby blocking the release of inflammatory cytokines, which is conducive to the survival of *Salmonella* in the host (**Figure 5**) (91). Although we know that the inflammasome plays an important role in inducing pyroptosis, whether DinJ can induce GSDM protein family activation remains to be verified.

## 5 The significance of pyroptosis in the salmonellosis

### 5.1 Pyroptosis to intestinal inflammatory disorders induced by *Salmonella*

Salmonellosis is one of the most common foodborne infections. *Salmonella* infection in humans and animals can cause intestinal damage, resulting in intestinal inflammation, more severe cases can lead to bacteremia. Studies have shown that pyroptosis is involved in a variety of diseases, such as ulcerative colitis. Pyroptosis plays an important central role in intestinal immune defense and pathology by regulating microbial infections and secretion of inflammatory factors, ROS production, or lysosomal damage (107). Researchers have established a model of *Salmonella* infection in mice and found that activation of the canonical and non-canonical inflammasome pathways can control *Salmonella* pathogen burdens and IEC shedding in the mice intestine, which highlights the importance of IEC pyroptosis as a host defense mechanism (10, 108). Evidence from other studies suggested that NAIP/NLRC4 inflammasome can protect mice from a TNF-driven inflammatory response during *Salmonella* infection (109). In other words, NAIP/NLRC4 inflammasome-mediated pyroptosis eliminates the replicative niche for *Salmonella.* In addition, other studies have shown that GSDMD restricts *Salmonella* Typhimurium loads in the gut tissue and systemic organs, controls gut inflammation kinetics, and prevents epithelium disruption by 72 h of the infection (110).

The precise identification and suppression of pyroptosis are essential for the survival and proliferation of *Salmonella* within the host. For example, as we mentioned earlier, the SopB of *Salmonella* evades recognition and clearance by B cells, a type of immune cell, by inhibiting pyroptosis (55, 56). The same study showed the importance of SpvC for bacterial dissemination in mice and damage to secondary tissues, such as spleen and liver, during infection (75). These findings indicate that downregulation of pyroptosis by the virulence factor is essential for bacterium’s survival and reproduction within the host. In conclusion, pyroptosis exhibits both protective and detrimental effects during *Salmonella* infection. It is imperative to embrace a dialectical perspective on this matter. Interventions that are both targeted and selective ought to be grounded in the pathogenic mechanisms of *Salmonella*.

### 5.2 Potential mechanisms underlying pyroptosis in the intestinal damage induced by *Salmonella*

Intestinal homeostasis serves as the cornerstone for sustaining overall health. Disruption of intestinal homeostasis due to various factors can result in a range of diseases, with inflammasomes, pyroptosis, and their associated signaling pathways playing crucial roles in the maintenance of intestinal equilibrium. Short-chain fatty acids (SCFAs) generated by gut microbiota can modulate immune responses. Hockenberry et al. found that SCFAs could decrease population-level T3SS-1 expression of *Salmonella* (111). Other studies have demonstrated that SCFAs enhance inflammasome activation by binding to the ASC PYRIN domain in macrophages, thereby promoting heightened inflammasome activity that suppresses *Salmonella* survival through pyroptosis and facilitates neutrophil recruitment to bolster bacterial elimination, ultimately inhibiting systemic dissemination within the host (112). These findings have implications that SCFA levels and their dynamics play a crucial role in preventing *Salmonella* colonization of the gut and bacterial elimination. Although our current understanding of the impact pyroptosis on intestinal microbiota within *Salmonella* infection is limited, it remains an area of ongoing research. As a result, this enhances our understanding of the intricate mechanisms by which *Salmonella* orchestrates pyroptosis during infection and unveils groundbreaking insights for prospective therapeutic strategies aimed at combating salmonellosis.

## 6 Conclusions and future perspectives

**Figure 6:**
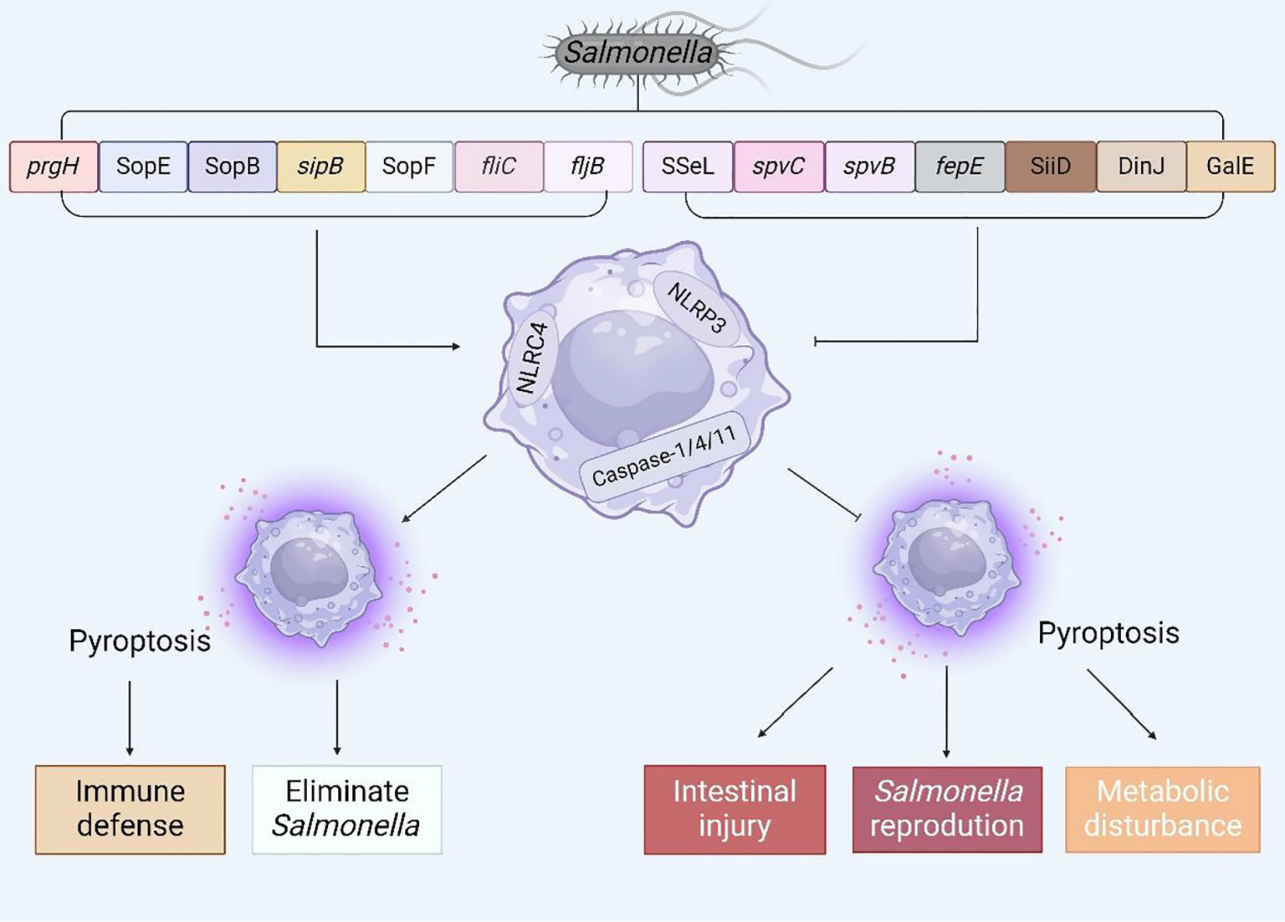
The interaction between virulence factors and effector proteins of *Salmonella* and pyroptosis. Summarize the virulence genes and effector proteins of *Salmonella* that can cause pyroptosis, and analyze the possible consequences of regulating pyroptosis.

In recent years, an in-depth investigation into pyroptosis has elucidated our comprehension of its molecular underpinnings and its crucial function in the context of *Salmonella* infection. To a certain extent, pyroptosis can endow the host with resistance to *Salmonella* invasion; nonetheless, inversely, *Salmonella* has the capability to inhibit host pyroptosis, thereby facilitating its colonization and enhancing its survival within the host’s microenvironment. Given the dual nature of this phenomenon, the objective of this article is to summarize the complex mechanisms by which *Salmonella* virulence factors or effector proteins regulate host pyroptosis (**Figure 6**) and to propose several potential therapeutic approaches for future interventions aimed at countering *Salmonella* infections.

Firstly, from the perspective of *Salmonella* pathogenicity, Zigangirova and his colleagues have discovered a novel small molecule inhibitor, Fluorothiazinon (formerly known as CL-55), which effectively reduces the replication and proliferation of *Salmonella* by inhibiting its T3SS, while not hindering it *in vitro* growth (113). The small molecule inhibitor quercitrin has demonstrated a significant capacity to repress the expression of SPI-1 genes and associated effectors, thereby impairing T3SS functionality and substantially reducing both *Salmonella* colonization and cecal pathological damage in murine models (114). It would be judicious to consider the development of inhibitors that are specifically tailored to target the secretion system of *Salmonella*, thereby interrupting the discharge of effector proteins pivotal to circumventing pyroptosis, and consequently, curbing bacterial proliferation. Moreover, as previously encapsulated, the excision of specific virulence genes in *Salmonella* may result in either an upsurge or a suppression of pyroptosis in the host, thereby affecting its viability within the host organism. This revelation also implies that the creation of novel attenuated vaccines targeting *Salmonella* could prove to be a critical tactic in the future prevention and management of infections attributable to this bacterium.

Secondly, from the vantage point of pyroptosis, we may consider the strategy of achieving pathogen elimination by selectively stimulating pyroptosis during the infectious process. For example, MCC950, a specific small molecule inhibitor, has been documented to markedly suppress inflammasome activation in diverse disease models (115). Traditional Chinese medicinal compounds, such as Licochalcone B (LicoB), Oridonin (Ori), and Helenin, act as targeted inhibitors of the NLRP3 inflammasome by interfering with the interaction between NLRP3 and NEK7, thus inhibiting both the assembly and activation of this inflammasome complex (116–118). Investigators have shown that Coptisine and Compound parthenolide potently inhibit NLRP3 inflammasome activation and NF-κB signaling in macrophages by downregulating caspase-1 protease activity, thereby preventing the occurrence of inflammatory cytokine storms (119, 120). These findings suggest that both traditional Chinese medicine monomers and natural small molecule compounds, which are directed at the regulation of inflammasomes and pyroptosis, may pave new pathways for the prophylaxis and management of *Salmonella* infections, as well as additional bacterial infections, in future therapeutic endeavors.

Finally, we consider from the perspective of the host. The metabolic reprogramming of immune cells is instrumental in orchestrating the inflammatory response, activating inflammasomes, and facilitating pathogen persistence within the host. *Salmonella* augments its virulence by prompting metabolic changes in host macrophages and harnessing the resultant glucose accumulation as a substrate for intracellular proliferation (121). Additional studies have underscored the notion that various metabolic pathways converge to serve as potent regulators of the NLRP3 inflammasome, which intricate structure that initiates pyroptosis (122). By manipulating metabolic pathways or metabolites to facilitate pyroptosis, leveraging the pathogen’s metabolic reprogramming traits or those of the infected host during infection, it may be feasible to regulate pathogen infection, thereby presenting novel avenues for the treatment of salmonellosis (123). Elucidating the role of metabolic reprogramming in pathogen-induced pyroptosis, especially within the context of *Salmonella* infection, requires further investigation and a deeper level of understanding. A more profound grasp of metabolic reprogramming and the mechanisms of cell pyroptosis could pave the way for the development of innovative concepts and strategies to combat *Salmonella*-related maladies in the future.

Although our comprehension of the virulence factors and effector proteins in *Salmonella* has reached an extensive level, a deficit persists in our knowledge regarding the intrinsic pathogenic mechanisms, necessitating a continued pursuit of understanding and investigation. The regulation of pyroptosis by other virulence factors or effector proteins in *Salmonella* remains largely elusive, underscoring the need for additional research. Elucidating the regulatory mechanisms through which *Salmonella* virulence factors and effector proteins trigger pyroptosis could pave the way for novel concepts and strategies in the clinical prevention of *Salmonella* infections and the treatment of associated diseases.

## Glossary

RCD: regulated cell death
PCD: programmed cell death
T3SS: the type III secretion system
T1SS: the type I secretion systems
GSDM: gasdermin protein family
LPS: lipopolysaccharide
GSDMD: gasdermin D
GSDME: gasdermin E
ELANE: neutrophil-specific serine protease neutrophil elastase
PRRs: pattern recognition receptors
ASC: apoptosis-associated speck-like protein containing a caspase recruitment domain
PYD: pyrin domain
CARD: caspase recruitment domain
NLR: nucleotide-binding domain and leucine-rich-repeat-containing protein
ALR: AIM2-like receptor protein
PAMPs: pathogen-associated molecular patterns
DAMPs: damage-associated molecular patterns
NLRP3: nucleotide-binding oligomerization domain-like receptor family, pyrin domain-containing 3 inflammasome
NAIP: NLR apoptosis inhibitory protein
OMVs: Outer membrane vesicles
SPIs: Salmonella pathogenicity islands
HGT: horizontal gene transfer
IECs: intestinal epithelial cells
JNK: the c-Jun N-terminal kinase signaling pathway
SPtA: Salmonella Paratyphi A
SCVs: Salmonella-containing vesicles
PDK1: phosphoinositide dependent protein kinase-1
RSK: Ribosomal S6 kinase
MLKL: mixed lineage kinase domain-like protein
SseL: Salmonella secreted factor L
MAPK: mitogen-activated protein kinase signaling pathway
SOCS-3: suppressor of cytokine signaling
ROS: reactive oxygen species
Spv: Salmonella plasmid virulence
CXCL2: C-X-C motif chemokine ligand 2
CXCL3: C-X-C motif chemokine ligand 3
CXCR2: C-X-C motif chemokine receptor 2
IPAF: Nod-like receptor ICE protease-activating factor
BMDMs: Bone Marrow-Derived Macrophages
TA: type II toxin-antitoxin system
SCFAs: Short-chain fatty acids
LicoB: Licochalcone B
Ori: Oridonin
